# Molecular characterization of the *Corynebacterium pseudotuberculosis hsp60-hsp10 *operon, and evaluation of the immune response and protective efficacy induced by *hsp60 *DNA vaccination in mice

**DOI:** 10.1186/1756-0500-4-243

**Published:** 2011-07-20

**Authors:** Marcilia P Costa, John A McCulloch, Síntia S Almeida, Fernanda A Dorella, Cristina T Fonseca, Diana M Oliveira, Maria FS Teixeira, Ewa Laskowska, Barbara Lipinska, Roberto Meyer, Ricardo W Portela, Sérgio C Oliveira, Anderson Miyoshi, Vasco Azevedo

**Affiliations:** 1 Departamento de Biologia Geral. Instituto de Ciências Biológicas. Universidade Federal de Minas Gerais. Av. Antonio Carlos, 6627 - Pampulha, CP 486, CEP 31.270-901. Belo Horizonte- MG, Brazil; 2 Faculdade de Medicina Veterinária, Núcleo de Genômica e Bioinformática. Universidade Estadual do Ceará. Av. Paranjana, 1700 - Itaperi, CEP 60.740-000. Fortaleza-CE, Brazil; 3 Departamento de Genética. Instituto de Ciências Biológicas. Universidade Federal do Pará, R. Augusto Corrêa, 01 - Guamá, CP 8607, CEP 66.075-900. Belém-PA, Brazil; 4 Fundação Oswaldo Cruz. Centro de Pesquisa René Rachou, Av. Augusto Lima, 1715 - Barro Preto, CEP 30.190-002. Belo Horizonte-MG, Brazil; 5 Department of Biochemistry. University of Gdansk, ul. Bażyńskiego 1a, Postal Code 80-952. Gdańsk, Poland; 6 Departamento de Ciências da Biointeração. Instituto de Ciências da Saúde. Universidade Federal da Bahia, Av. Reitor Miguel Calmon, s/n - Vale do Canela, CEP 40110100. Salvador-BA, Brazil

## Abstract

**Background:**

Heat shock proteins (HSPs) are important candidates for the development of vaccines because they are usually able to promote both humoral and cellular immune responses in mammals. We identified and characterized the *hsp60-hsp10 *bicistronic operon of the animal pathogen *Corynebacterium pseudotuberculosis*, a Gram-positive bacterium of the class *Actinobacteria*, which causes caseous lymphadenitis (CLA) in small ruminants.

**Findings:**

To construct the DNA vaccine, the *hsp60 *gene of *C. pseudotuberculosis *was cloned in a mammalian expression vector. BALB/c mice were immunized by intramuscular injection with the recombinant plasmid (pVAX1/*hsp60*).

**Conclusion:**

This vaccination induced significant anti-hsp60 IgG, IgG1 and IgG2a isotype production. However, immunization with this DNA vaccine did not confer protective immunity.

## Findings

*Corynebacterium pseudotuberculosis *is a facultative, intracellular, Gram-positive bacterium of the class *Actinobacteria*, which also includes the genera *Mycobacterium, Nocardia *and *Rhodococcus*. The latter three genera, together with *Corynebacterium*, form a group of potentially pathogenic species termed the CMN group. *Corynebacterium pseudotuberculosis *is the etiological agent of caseous lymphadenitis (CLA), or cheesy gland, which affects small ruminants (sheep and goats) and occasionally other hosts. This chronic disease is pathognomonically characterized by the formation of suppurative abscesses in superficial and internal lymph nodes. In severe cases, these abscesses are also found in internal organs, such as the lungs, kidneys, liver and spleen, characterizing visceral CLA [1]. The economic relevance of CLA, its widespread occurrence, and a lack of knowledge regarding its molecular mechanisms of virulence, have prompted the investigation of its pathogenesis with the aim to develop efficient treatment strategies against this disease [2].

Chemotherapeutic treatment of CLA is difficult because the bacteria are shielded within granulomas, where they are relatively protected from antibiotic drugs [3]. Therefore, attempts to control CLA usually entail immunoprophylaxis by vaccination. Various strategies have been used for developing vaccines against CLA, including the use of inactivated or attenuated *C. pseudotuberculosis *strains [4,5], fractions of bacterial cells containing bacterial antigens, antigens from culture supernatants, and DNA vaccines [6]. None of the currently commercially available vaccines for *C. pseudotuberculosis *provide effective protection against CLA [7].

Heat shock proteins (HSPs), or molecular chaperones, are traditionally classified according to their molecular weight; they are highly conserved proteins, abundantly expressed in eukaryotic and prokaryotic organisms [8]. These proteins are expressed in unstressed cells at low levels, and play an important role in cell survival both under normal physiological conditions, during various phases of the cell cycle, cellular differentiation and growth, and under stress conditions, such as heat shock [9]. Heat shock proteins are considered immunologically important due to the fact that they are recognized by the host in bacterial, fungal, and parasitic infections and are therefore capable of inducing strong humoral and cellular immune responses in mammals [10].

Several studies have shown that these versatile proteins can be used as antigens for the development of vaccines against diseases. In the case of infectious diseases, HSPs could play a dual role in vaccine development. Pathogen-derived HSPs can be used as vaccine antigens, and host- and pathogen-derived HSPs can be used as adjuvants [11,12]. Strategies to more effectively induce immunity with HSPs include the use of DNA vaccines. HSP-based DNA vaccines have been effective in several immunization trials against *Mycobacterium spp*. infection [12].

The Hsp60 protein of *C. pseudotuberculosis*, using a protein subunit as immunogen against CLA, failed to confer protection against infection with *C. pseudotuberculosis *in mice [13]. Using an alternative strategy, we assessed the feasibility of using DNA encoding *hsp60 *for protection against experimental challenge with *C. pseudotuberculosis*.

## Methods

### Bacterial strains, growth conditions and plasmids

All bacterial strains, plasmids and PCR primers used in this study are listed in Table 1. *Escherichia coli *TOP10 was grown in Luria-Bertani broth (LB, Difco Laboratories, Detroit, USA) at 37°C with stirring for 18 h. Plasmid-containing transformants were selected by the addition of ampicillin (Invitrogen, San Diego, CA) and X-Gal (Invitrogen, San Diego, CA) to the media. The supplement concentrations were ampicillin (100 μg/mL) and X-Gal (40 μg/mL).

**Table 1:** Strains and plasmids used in this study.

Description or sequence	
***Strain***	

*Corynebacterium pseudotuberculosis *biovar *ovis*	T1 (virulent strain isolates from goats; obtained from the Universidade Federal da Bahia, UFBA, Brazil). MIC-6 (virulent strain isolate from goats; obtained from the Laboratório de Genética Celular e Molecular -LGCM, UFMG, Brazil).

*E. coli*	TOP10 [F- mcrA Δ(mrr-hsdRMS-mcrBC) φ80lacZΔM15 ΔlacX74 nupG recA1 araD139 Δ(ara-leu)7697 galE15 galK16 rpsL(StrR) endA1 λ-] Invitrogen. B178 *groEL44 *mutant (Δg*roEL44*) characterized by Zeilstra-Ryalls *et al *[19].

***Plasmid***	

pTopo^® ^Cloning vector	Cloning vector - ColE1/Ap^r ^- Invitrogen.

pVAX1^© ^Vector	Eukaryotic expression vector - pUC/Km^r ^- Invitrogen.

pTopo/*hsp*60	Cloning vector with the *C. pseudotuberculosis hsp60 *gene inserted in the *Bam*HI and *Hind*III restriction sites of the vector.

pProEx-Hta/*hsp*60	Prokaryotic expression vector containing the *C. pseudotuberculosis hsp60 *gene [13].

pVAX1/*hsp*60	Eukaryotic expression vector containing the *C. pseudotuberculosis hsp60 *gene.

Isopropyl-β-D-thiogalactopyranoside (IPTG) was used at a final concentration of 1 mM in complementation experiments.

*Corynebacterium pseudotuberculosis *biovar *ovis *strain T1 was aerobically grown in brain heart infusion broth (BHI, Acumedia Manufacturers, Inc., Baltimore, MD, USA) and on 1.5% (w/v) BHI agar plates at 37°C for 48-72 h [14].

### DNA isolation

All DNA templates were prepared with genomic DNA isolated by collecting a bacterial cell pellet from culture, as previously described [14]. Briefly, an aliquot of 20 mL from a 48 to 72 h culture was centrifuged at 4°C and 2000 × *g *for 20 min. Cell pellets were resuspended in 1 mL of Tris/EDTA/RNase [10 mM Tris/HCl (pH 7.0), 10 mM EDTA (pH 8.0), 300 mM NaCl, 50 mg RNaseA mL^-1^] and centrifuged again under the same conditions. Supernatants were discarded and the pellets were resuspended in 1 mL of TE/lysozyme. Samples were then incubated at 37°C for 30 min; 30 μL of 30% (w/v) sodium N-lauroylsarcosine (sarcosyl) were added, and the mixture was incubated for 20 min at 65°C, followed by incubation for 5 min at 4°C. DNA was purified using phenol/chloroform/isoamyl alcohol and precipitated with ethanol. DNA concentrations were determined spectrophotometrically.

### PCR amplification and cloning

PCR reagents, restriction endonucleases, and ligation reagents used in this study were all purchased from Invitrogen, San Diego, CA. Both genes were amplified by PCR using genomic DNA of *C. pseudotuberculosis *as a template, with primers that were designed based on the DNA sequence of paralogs of *C. diphtheriae *NCTC 13129 (NCBI Acc. GeneID: 2648771 and NCBI Acc. GeneID: 2648772). The *C. pseudotuberculosis hsp60 *gene was amplified by PCR using the following primers: 5'- GATGGCAAAGCTGATTGCA -3' (sense orientation) and 5'- TTAGTGGTGGTGATGGTG -3' (antisense orientation). The PCR assays were carried out in a final reaction volume of 50 μL, containing 20 ng genomic DNA, 2 μM of each of the primers, 1 × PCR Buffer II and 1 U AccuPrime taq DNA polymerase. Amplification was run in a thermal cycler (PTC-100, MJ Research, Inc.) as follows: one cycle of 95°C for 5 min; 29 cycles of 95°C for 1 min, 50°C for 40 s, and 68°C for 2 min 30 s; and a final extension step at 68°C for 7 min. The *hsp60 *fragment was purified from bands in 1.0% (w/v) agarose gels using the Consert TM Rapid Gel Extraction System kit (Gibco-BRL, Gaithersburg, MD, USA). The retrieved DNA fragment was then ligated into the pCR^®^2.1-TOPO^® ^vector, as described in the manufacturer's protocol. The recombinant plasmid (pCR^®^2.1-TOPO^®^/*hsp60*) was then introduced into competent *E. coli *TOP10 cells, and single recombinant colonies were selected. Plasmid DNA was isolated from cells by the alkaline lysis method [15]. The presence of the inserted DNA fragment was confirmed by sequencing, using the DYEnamic ET Dye Terminator kit (Amersham Biosciences, Piscataway, NJ, USA).

To verify operon structure, the *hsp10 *gene and intergenic regions of the operon were amplified using the following primer pair: 5'- GTGGCTAACGTCAATATCAAGCC -3' (sense orientation) (designed based on the DNA sequence of paralogs of *C. diphtheria *NCTC 13129) and 5'- CTTCAGGATGCCTTCACGGG -3' (antisense orientation) (designed based on the DNA sequence of the *C. pseudotuberculosis hsp60 *gene). The PCR assays were carried out in a final reaction volume of 50 μL containing 20 ng genomic DNA, 2 μM of each of the primers, 1 × PCR Buffer II and 1 U AccuPrime taq DNA polymerase. Amplification was performed with a thermal cycler (PTC-100, MJ Research, Inc.) as follows: one cycle of 95°C for 3 min; 29 cycles of 95°C for 1 min, 50°C for 40 s, and 72°C for 1 min 30 s; and a final extension step at 72°C for 5 min. The amplicon containing *hsp10 *was also purified, cloned, transformed, and sequenced as described above.

### Bioinformatics and comparative genomics

Sequence homology analysis was carried out against nucleotide and protein databases available in GenBank http://www.ncbi.nlm.nih.gov/, using the Blast tool http://www.ncbi.nlm.nih.gov/BLAST/ [16]. Multiple sequence alignments and analysis were performed using the ClustalW algorithm http://www.ebi.ac.uk/clustalw/. The predicted amino acid sequences were analyzed to identify conserved motifs, using the Conserved Domains program http://www.ncbi.nlm.nih.gov/Structure/cdd/wrpsb.cgi and the ExPaSy ScanProsite program http://www.expasy.org/tools/scanprosite/. For three-dimensional modeling, we employed MODELLER http://salilab.org/modeller/modeller.html [17]. The isoelectric point (Ip) and molecular weight (Mw) were predicted with software found on the ExPaSy website http://web.expasy.org/compute_pi/ [18].

### Functional characterization of the *C. pseudotuberculosis hsp60 *gene

To examine the functional activity of the *C. pseudotuberculosis hsp60 *gene, *E. coli *B178*groEL44 *mutant (Δ*groEL44*) was transformed with a pProEx-Hta/*hsp60 *plasmid constructed by Pinho *et al *[13]. The *E. coli *Δ*groEL44 *mutant is a strain that bears a temperature-sensitive allele of *groEL*, namely *groEL44*. This mutant strain is unable to propagate bacteriophages γ, T4 or T5 [19]. Two phenotypes were analyzed in this complementation assay: heat stress resistance and inability of *γc*Ib2 phage to grow without the protein coded by the *hsp60 *(*groEL*) gene, as described by Kumar *et al *[20], with adaptations for use in our study. First, the empty vector (pProEx-Hta) and the recombinant vector (pProEx-Hta/*hsp60*) were transformed into the *E. coli *wild-type B178 and Δ*groEL44 *strains. To investigate the effect of temperature on protein expression, the bacteria were grown in LB broth containing ampicillin and IPTG. The protein was visualized in an SDS-polyacrylamide gel [21]. Aliquots of 3 μL of the induced cultures were plated on LB, with and without IPTG, in dilutions from 10^-1 ^to 10^-6^, and the plates were incubated at 30 and 42°C. Finally, to assess the growth of the *γc*Ib2 phage, *E. coli *Δ*groEL44 *was transformed with the empty and recombinant vectors (*groEL44*[pProEX-Hta] and *groEL44*[pProEx-Hta/*hsp60*]), while the wild-type *E. coli *was transformed with the empty vector (B178[pProEX-Hta]). Dilutions of the phage (10^-1^, 10^-2^, 10^-3 ^and 10^-4^) were made. Aliquots of the dilutions were plated on LB containing these transformants, with and without IPTG, and the plates were incubated at 30°C for 18 h.

### Plasmid construction for the DNA vaccine

The *hsp60 *gene was amplified using primers containing restriction sites and the Kozak consensus sequence within the primer: 5'- AAGCTTACCATGGCAAAGATTGCATT - 3' (sense orientation) and 5'- GGATCCTTAGTGGTGGTGATGGTG - 3' (antisense orientation), which include the *Hin*dIII and *Bam*HI sites (underlined), respectively. The *hsp60 *gene was amplified, cloned and transformed as described above. The recombinant plasmid (pCR^®^2.1-TOPO^®^/*hsp60*) and the eukaryotic expression vector (pVAX1^©^) were submitted to *Hin*dIII/*Bam*HI double digestion. The digested samples were analyzed by 1% agarose gel electrophoresis. DNA fragments were purified as described above. The purified DNA fragments were then subcloned into the pVAX1 vector. The components and conditions of the ligation reaction were according to the manufacturer's instructions. The presence and the correct size of the insert were confirmed by restriction enzyme digestion (*Eco*RI*/Hin*dIII). Both the empty vector (control) and the recombinant vector (pVAX1/*hsp60*) were introduced into competent *E. coli *TOP10, and single recombinants were selected. Control and pVAX1/*hsp60 *vectors were isolated and purified using the EndoFree Plasmid Giga kit (Qiagen, Valencia, CA, USA). DNA concentration and purity were determined by absorbance at 260 and 280 nm.

### Immunization and challenge

BALB/c mice (6-8 weeks old) were divided into two groups of five mice each. Mice were pretreated with 10 μM cardiotoxin five days before the first DNA immunization, as previously described [22]. Each animal was immunized by injecting the quadriceps muscle with four doses of 100 μg at 15-day intervals, with an empty vector (pVAX1; control) or a recombinant vector pVAX1/*hsp60*. Blood samples from the mice were collected by retro-orbital plexus puncture 15, 30, 45 and 60 days after the first immunization. All mice were challenged intraperitoneally 21 days after the fourth vaccination with an infectious dose of 1 ± 10^6 ^CFU of the MIC-6 strain of *C. pseudotuberculosis*. After challenge, the mice were monitored daily and the protective effect of the DNA vaccine was assessed by evaluation of the survival rate of the immunized animals. This experiment was performed twice to confirm the results.

### Determination of antibody levels

To examine the humoral responses induced by pVAX1/*hsp60*, the levels of specific anti-*hsp60 *IgG, IgG1 and IgG2a isotypes in individual mouse sera were determined by ELISA. Flat-bottomed 96-well plates were coated with the *C. pseudotuberculosis *Hsp60 protein in 0.05 M carbonate/bicarbonate buffer, pH 9.6, (5 μg/mL per well) at 4°C for 18 h. The plates were washed three times with PBS-0.05% T20 (1X PBS, pH 7.4, 0.05% Tween 20) and then blocked with PBS-0.05% T20 containing 10% bovine serum albumin - BSA (250 μL per well) at room temperature for 2 h. Plates were then washed three times with PBS-0.05% T20 before addition of a 1 in 100 serum dilution (100 μL per well) from immunized mice. The plates were then incubated at room temperature for 1 h, and subsequently washed three times with PBS-0.05% T20. Afterwards, 100 μL of peroxidase-conjugated anti-mouse IgG (1 in 5000), IgG1 (1 in 5000) and IgG2a (1 in 2000) antibodies were added per well (Southern Biotechnology, Birmingham, AL), and the plates were incubated for 1 h. The reaction was developed by adding 200 pmol orthophenyldiamine (Sigma-Aldrich, Bornem, Belgium) for 15 min and stopped by the addition of 50 μL of 6% H_2_SO_4 _to each well. The plates were read at 492 nm with an automatic microplate reader (Bio-Rad, Hercules, CA).

### Statistical analysis

All data were expressed as means ± standard deviation (S.D.) and analyzed using GraphPad Prism, version 4.03, for Windows (GraphPad Software, San Diego, CA). Statistical differences between groups were identified using one-way ANOVA. A one-tailed Student's *t*-test was used to determine if there were significant differences between the experimental and control groups. A *P *value of 0.05 or less was considered significant.

## Results

### Isolation and characterization of the *C. pseudotuberculosis hsp60 and hsp10 *genes

**Figure 1:**
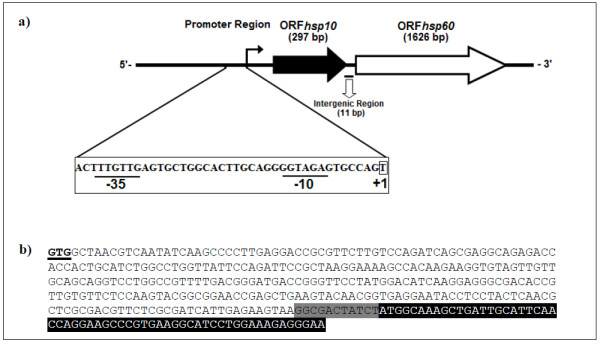
**Characterization of the *C. pseudotuberculosis *bicistronic *hsp60-hsp10 *operon**. In *C. pseudotuberculosis*, the *hsp10 *gene maps upstream of the *hsp60 *gene, and the stop codon of *hsp10 *is separated from the start codon of *hsp60 *by a 11 bp intergenic region. The putative -10 and -35 hexamers and of the start point of transcription (+1) are indicated; b) Nucleotide sequences of the *C. pseudotuberculosis hsp60-hsp10 *operon organization. The initial codon of *hsp10 *gene is underlined; the intergenic space (11 bp) between *hsp60-hsp10 *genes is shaded grey; and the initial sequence of the *hsp60 *gene is shaded black.

**Figure 2:**
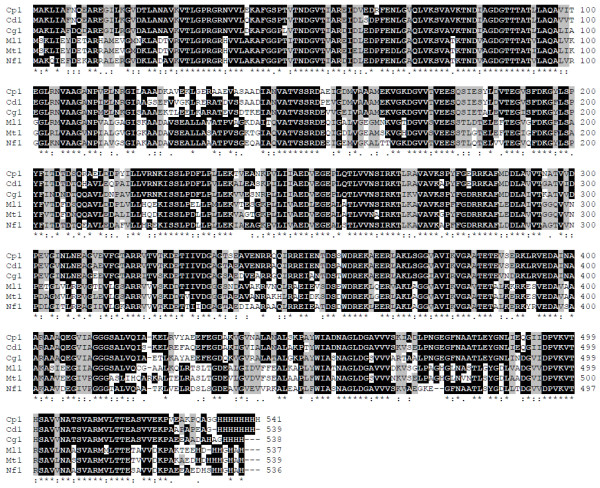
**Comparative analyses of Hsp60 homologues**. Multiple alignment of the amino acid sequences of 60 kDa chaperonin 1 gene products from *C. pseudotuberculosis *strain T1 (Cp1) [NCBI Acc. No. AAV48830.1] compared to Hsp60 from different organisms, including *C. diphtheriae *NCTC 13129 (Cd1) [NCBI Acc. No. NP_938952.1], *C. glutamicum *ATCC 13032 (Cg1) [NCBI Acc. No. NP_599834.1], *M. leprae *TN (Ml1) [NCBI Acc. No. NP_301373.1], *M. tuberculosis *H37Rv (Mt1) [NCBI Acc. No. NP_217934.1] and *N. farcinica *IFM 10152 (Nf1) [NCBI Acc. No. YP_117096.1]. Shaded areas in black indicate identical amino acids, and with gray background indicate similar amino acid residues. The gaps (-), identical (*) and similar (. or:) amino acid residues also are indicated.

**Figure 3:**
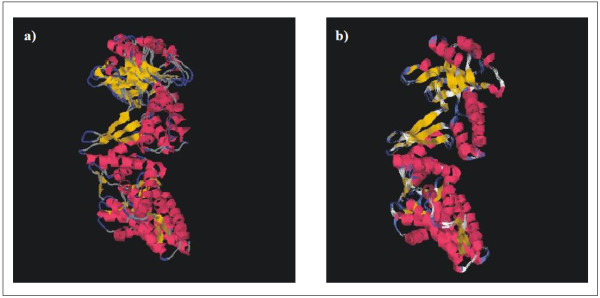
**Predictive 3D models of Hsp60**. Hsp60 from *C. pseudotuberculosis *(60 kDa chaperonin 1 gene, AAV48830.1); b) Hsp60 from *E. coli*, created with MODELLER, based on PDB related structures (1GR5, 1IOK, 1KP8, 1PCQ, 1SJP and 1WE3), as well as on combinatorial modeling. 3D structures are displayed in colored cartoon representations visualized with CHIME http://www.mdl.com. The α-helix and β-pin (loop) repeats of both structures are marked with the same colors.

The *C. pseudotuberculosis hsp60 *gene was amplified by PCR using primers that were designed based on the genome of *C. diphtheriae*, because of the phylogenetic proximity between these two species. The full lengths of the DNA sequences that were amplified were 1,626 (*hsp60*) and 297 (*hsp10*) nucleotides. The nucleotide sequences of the *C. pseudotuberculosis *strain T1 60 kDa chaperonin and 10 kDa chaperonin GroES coding sequences were deposited in GenBank under accession numbers AY781285 and DQ869271, respectively. These two genes are separated by a small sequence of 11 bp (Figure 1). In order to obtain information on similarity with genes of other species, the corresponding sequences were subjected to BlastN. Similarity searches of nucleotide sequences of both genes revealed significant identity with other *Corynebacterium *species. The *hsp60 *gene was predicted to encode a putative protein consisting of 541 amino acid residues, with a predicted molecular weight (MW) of 57.4 kDa and a theoretical isoelectric point (Ip) of 4.91. The *hsp10 *gene was predicted to encode a polypeptide of 98 amino acids, with a MW of 10.6 kDa and an Ip of 4.49. In order to search for the putative identities and functions of these genes, the sequences were subjected to BlastX searches against the protein database of GenBank. The putative Hsp60 protein displayed greater similarity to the Hsp60 protein of *C. diphtheriae *(89%) and *C. glutamicum *(82%), when compared to those of *Nocardia *(66.9%) and *Mycobacterium *(61%). The comparative alignment of primary amino acid sequences of Hsp60 homologues can be observed in Figure 2. The Hsp60 putative protein shows a motif characterized by a histidine-rich C-terminal. In order to compare the structure of Hsp60 protein of *C. pseudotuberculosis *with that of other chaperonins, a three-dimensional (3D) model was predicted based on PDB-related structures (Figure 3). The protein was found to have high sequence similarity (54% identity) with chaperonins of other bacteria.

### Functional characterization of *hsp60*

**Figure 4:**
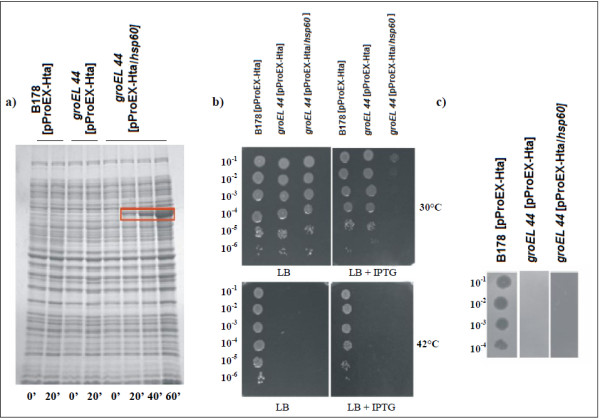
**Expression of *C. pseudotuberculosis *Hsp60 protein and complementation assays**. a) Expression of *C. pseudotuberculosis *Hsp60 protein in *E. coli groEL44 *mutant transformed with the recombinant vector pProEx-Hta:*hsp60 *(*groEL44*[pProEx-Hta/*hsp60*]); *E. coli *wild-type and mutant transformed with the empty vector (B178[pProEX-Hta] and *groEL44*[pProEX-Hta], respectively) were used as negative controls. Expression of recombinant protein was analyzed at 0, 20, 40 and 60 min after induction with IPTG. The rectangle indicates the protein expressed; b) Complementation assay after thermal stress. The growth of *E. coli *wild-type (B178[pProEX-Hta]), *groEL44 *mutant (*groEL44*[pProEX-Hta] and *groEL44*[pProEx-Hta/*hsp60*]) were evaluated. Cultures were serially diluted (10^-1 ^to 10^-6^), spotted onto the surface of LB agar plates (with and without 1 mM IPTG), and incubated at 30 or 42°C for 18 h; c) Complementation analysis of growth of the *γc*Ib2 phage in *E. coli *g*roEL44 *mutant. The growth of the phage was evaluated in *E. coli *wild-type (B178[pProEX-Hta]) and *groEL44 *mutant (*groEL44*[pProEX-Hta] and *groEL44*[pProEx-Hta/*hsp60*]). The cultures induced with IPTG, plated at the dilutions from10^-1 ^to 10^-4^, were incubated at 30°C for 18 h.

The GroEL mechanism is universally conserved in prokaryotic species; consequently, paralogous copies of GroEL may result in redundancy of chaperonin function in these organisms. In *E. coli*, the development of phage lambda (γ) requires a functional GroEL/S system. GroEL was originally identified as the host factor responsible for phage γ capsid protein assembly and was subsequently shown to be essential for cell viability [20]. We used a complementation test to functionally characterize the *hsp60 *gene (*groEL *gene) of *C. pseudotuberculosis *(Figure 4). Two *E. coli *strains were used for this purpose: the wild-type B178 strain and a Δ*groEL44 *mutant. We observed that the mutant transformed with the pProEx-Hta/*hsp60 *vector only grew at the 10^-1 ^dilution, when incubated at 30°C. When incubation was at 42°C, bacterial growth was not observed (Figure 4b). The Δ*groEL44 *mutant, incubated at 30°C, was also not phenotypically restored in a phage experiment. The expression of *C. pseudotuberculosis hsp60 (groEL) *did not promote phage development (Figure 4c).

### Anti-Hsp60 antibody response and protective efficacy of the DNA vaccine in a murine model

**Figure 5:**
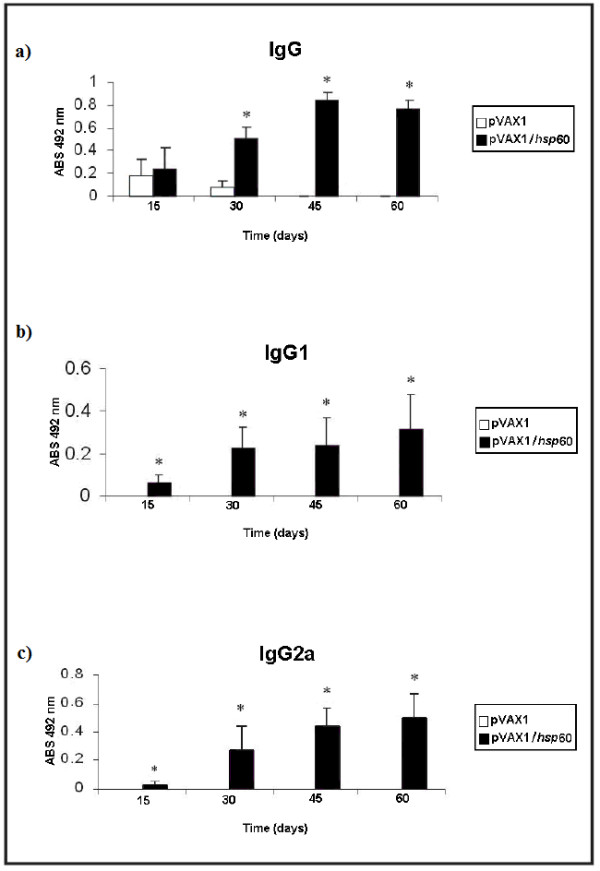
**The profile of total anti-Hsp60 IgG antibody response after DNA vaccination in mice**. a) IgG antibody titer; b) IgG1 isotype titer; c) IgG2a isotype titer. Groups of mice were immunized with the DNA vaccine as follows: intramuscular injection of vector pVAX1 and recombinant vector pVAX1/*hsp60*. Data are expressed as mean ± S.D. values. Results are representative of n = 5. Statistically significant differences between pVAX1/*hsp60 *and pVAX1 immunized mice are denoted by an asterisk (p < 0.05).

Vaccination with plasmid DNA encoding *hsp60 *induced significant levels of anti-Hsp60 IgG, IgG1 and IgG2a antibodies in BALB/c mice, after the second immunization, compared to levels in mice vaccinated with the empty vector (Figure 5). Increased antibody responses following boosting were observed for all the isotypes. The levels of IgG1 and IgG2a at 15, 30, 45 and 60 days after the first immunization and the IgG1/IgG2a ratio were recorded (Table 2). The results suggest a Th2-type immune response, induced by pVAX1/*hsp60 *vaccination, at 15 days after the first immunization, with a decrease of the IgG1/IgG2a ratio at 30, 45 and 60 days after the first immunization.

**Table 2:** IgG1 and IgG2a immune profile induced by vaccination with pVAX1/*hsp60 *or pVAX1 vectors.

Days ^a^	Groups				
	
	IgG1		IgG2a		IgG1/IgG2a ratio
		
	pVAX1/*hsp60*	pVAX1	pVAX1/*hsp60*	pVAX1	
15	0.06 ± 0.039^b^	0.002 ± 0.001	0.03 ± 0.011^b^	0.003 ± 0.002	2.00
30	0.23 ± 0.094^b^	0.003 ± 0.001	0.27 ± 0.149^b^	0.002 ± 0.001	0.85
45	0.24 ± 0.133^b^	0.004 ± 0.002	0.43 ± 0.114^b^	0.004 ± 0.001	0.56
60	0.32 ± 0.165^b^	0.003 ± 0.002	0.49 ± 0.160^b^	0.004 ± 0.003	0.65

^a ^Days after the first immunization.

^b ^Significantly different compared to mice immunized with the pVAX1 vector.

### Protection studies

DNA vaccine protection studies were carried out in mice to test the potential of *hsp60 *for this purpose. All the animals died 12 days after challenge with the wild-type *C. pseudotuberculosis *MIC-6 strain. The mice began to display clinical signs of morbidity three days after infection; during the first two weeks the animals showed cachexia, piloerection, cyanosis, hypothermia and ascitis, all characteristic signs and symptoms of a *C. pseudotuberculosis *infection.

## Discussion

This study was the first to characterize the immunogenic potential of the *hsp60 *DNA vaccine for protection against the veterinary pathogen *C. pseudotuberculosis*. The *hsp60 *DNA vaccine induced a cellular immune response but failed to confer protective immunity to the host, which corroborates a previous study that found that an Hsp60 protein subunit vaccine also did not confer protection [13].

### Characterization of the *hsp60-hsp10 *bicistronic operon

We found that in *C. pseudotuberculosis *these genes are arranged in a bicistronic *hsp60-hsp10 *operon, separated by a small sequence of 11 bp. The size and organization of the *hsp60 *gene in *C. pseudotuberculosis *(1,626 bp) was similar to those described for other bacterial species, such as *C. glutamicum *(1,617 bp) [23] and *R. equi *(1,623 bp) [24]. The *hsp10 *gene (297 bp) has a start codon (GTG), and the predicted molecular weight is 10.6 kDa. These characteristics are conserved in the *hsp10 *genes of other species [25]. Comparative DNA sequence analysis of the *hsp10 *and *hsp60 *genes showed significant similarity with genes of microorganisms with phylogenetic proximity, especially between two *hsp60 *paralogs (*groEL1 *and *groEL2*) within the *Corynebacterium *genus [26]. Alignment of amino acid sequences coded by *hsp60 *revealed a higher identity at the N- and C- terminal regions. According to Barreiro *et al *[26] some microorganisms have different functional motifs at the C-terminal ends of the proteins coded by *hsp60*; there is a string of histidines in *groEL*1, while *groEL*2 has a glycine-glycine-methionine (GGM) motif. This putative protein showed a motif containing eight histidine residues at the C terminus, which is characteristic of *hsp60 *paralog *groEL*1 protein in actinomycetes [27]. The predicted tertiary structure of the Hsp60 protein showed three functionally distinct domains that are very characteristic of chaperonins: an α-helical equatorial, a small intermediate, and a highly flexible apical domain [28].

The high level of similarity between *hsp60 *of *C. pseudotuberculosis *and those of other important pathogens, such as *M. leprae *and *M. tuberculosis*, is important because other studies have indicated that mycobacterial Hsp60 is a potential immunodominant target of the humoral and T-cell response in mice and humans [29]. Additionally, high sequence homology of HSPs between different species results in HSPs that have cross-reactive epitopes [30].

Despite high homology between *C. pseudotuberculosis hsp60 *and *E. coli GroEL*, our complementation assay failed to complement the *GroEL *defect in *E. coli*. There are several explanations for these results. First, the *hsp60 *expressed in *E. coli *may have improperly folded and thus lacked activity to complement *GroEL*. Second, since HSPs have been reported to adversely affect protein homeostasis and vital intracellular functions [11], overexpression of *hsp60 *may have reduced cell viability at all concentrations except at the 10^-1 ^dilution at 30°C. Third, protein-protein interaction between *E. coli GroEL *and *GroES *(the corresponding *hsp60 *and *hsp10 *paralogs) is also necessary for cell viability [31]. The *GroEL *mutant *E. coli *complemented with the *C. pseudotuberculosis hsp60 *gene did not grow, indicating that there may be little protein-protein interaction between the Hsp60 and *E. coli groES *proteins. Finally, lack of growth may indicate that the Hsp60 protein cannot function inside *E. coli *despite protein homology because species-specificity of chaperone proteins is complex and is not explained simply by protein homology [32], because the regulatory mechanism of the heat shock response differs among species [33].

*E. coli GroEL *is also required for the proper growth of bacteriophages, including capsid formation [27,34]. The inability of the *C. pseudotuberculosis hsp60 *gene to support the growth of γ phage in the *E. coli GroEL *mutant suggests that the Hsp60 protein does not perform the specific function of capsid formation inside *E. coli*, again possibly due to incorrect folding.

### Immune response to *hsp60 *DNA vaccine in mice

Due to the success of DNA-based vaccines using genes encoding HSPs to induce immunity against a variety of pathogens [24], we chose the *C. pseudotuberculosis hsp60 *gene for the development of a DNA vaccine. Vaccination with the recombinant *C. pseudotuberculosis hsp60 *antigen induced significant production of specific anti-Hsp60 antibodies. These data indicate that the DNA vaccine (pVAX1/*hsp60*) generated both IgG1 and IgG2a responses, when administered to BALB/c mice, however, with a tendency towards a Th1-type immune response after 30 days of the first immunization based on reduced IgG1/IgG2a ratio. Nevertheless, DNA immunization with pVAX1/*hsp60 *conferred no protection against challenge with the pathogen; it did not prevent infection.

The protective efficacy of DNA vaccines has been studied extensively for *Mycobacterium tuberculosis*. For example, a DNA vaccine encoding the *M. leprae hsp65 *induced protective immunity against tuberculosis challenge in a mouse model [35], and a DNA vaccine with the *M. avium hsp65 *plasmid elicited a strong protective immune response in lambs and protected against *M. avium *subspecies *paratuberculosis *infection [36]. However, other studies have shown that a high antibody response induced by DNA vaccines does not always result in protective immunity [24], as we also observed in our study.

In summary, intramuscular administration of *hsp60 *DNA vaccine in mice induced an immune response but failed to confer protection against infection with *C. pseudotuberculosis*.

## Competing interests

The authors declare that they have no competing interests.

## Authors' contributions

MPC, FAD, SA, JAM contributed to the design of the study and the data analysis, and wrote the manuscript. FAD and CTF performed the majority of experiments. EW and BL performed the functional characterization assay. DMO, MTST, RM, RWP, SCO, AM and VA were involved in the study design, management and coordination, and the drafting of the manuscript. The authors have read and approved the final draft of the manuscript.
